# Temperature is a key determinant of alpha- and beta-synuclein membrane interactions in neurons

**DOI:** 10.1016/j.jbc.2021.100271

**Published:** 2021-01-09

**Authors:** Nagendran Ramalingam, Ulf Dettmer

**Affiliations:** Ann Romney Center for Neurologic Diseases, Brigham and Women’s Hospital and Harvard Medical School, Boston, Massachusetts, USA

**Keywords:** Parkinson’s disease, α-synuclein, β-synuclein, membrane binding, sequential extraction, αS, α-synuclein, βS, β-synuclein, DIV, days *in vitro*, GAPDH, glyceraldehyde 3-phosphate dehydrogenase, HBSS, Hank’s balanced salt solution, kDa, kilodalton, LDS, lithium dodecyl sulphate, mAb, monoclonal antibody, min, minutes, MOI, multiplicity of infection, MW, molecular weight, pAb, polyclonal antibody, PBS, Phosphate-buffered saline, PFA, paraformaldehyde, RT, room temperature, TBST, Tris-buffered saline with Tween-20, wt, wild-type

## Abstract

Aggregation of α-synuclein (αS) leads to the hallmark neuropathology of Parkinson’s disease (PD) and related synucleinopathies. αS has been described to exist in both cytosolic and membrane-associated forms, the relative abundance of which has remained unsettled. To study αS under the most relevant conditions by a quantitative method, we cultured and matured rodent primary cortical neurons for >17 days and determined αS cytosol:membrane distribution *via* centrifugation-free sequential extractions based on the weak ionic detergent digitonin. We noticed that at lower temperatures (4 °C or room temperature), αS was largely membrane-associated. At 37 °C, however, αS solubility was markedly increased. In contrast, the extraction of control proteins (GAPDH, cytosolic; calnexin, membrane) was not affected by temperature. When we compared the relative distribution of the synuclein homologs αS and β-synuclein (βS) under various conditions that differed in temperature and digitonin concentration (200–1200 μg/ml), we consistently found αS to be more membrane-associated than βS. Both proteins, however, exhibited temperature-dependent membrane binding. Under the most relevant conditions (37 °C and 800 μg/ml digitonin, *i.e.*, the lowest digitonin concentration that extracted cytosolic GAPDH to near completion), cytosolic distribution was 49.8% ± 9.0% for αS and 63.6% ± 6.6% for βS. PD-linked αS A30P was found to be largely cytosolic, confirming previous studies that had used different methods. Our work highlights the dynamic nature of cellular synuclein behavior and has important implications for protein-biochemical and cell-biological studies of αS proteostasis, such as testing the effects of genetic and pharmacological manipulations.

αS has been implicated as a key pathogenic protein in both sporadic and familial PD (and related synucleinopathies) since its discovery as the first PD-associated gene product (1) and the major constituent of Lewy bodies (2), the hallmark cytopathology of synucleinopathies. Early characterizations of purified recombinant αS identified the protein to be soluble (3, 4), consistent with its immunogold-electron microscopical detection throughout cytoplasmic matrices in axon terminals (5). Subsequently, however, biophysical and biochemical studies concluded that αS can also bind to small unilamellar and multilamellar vesicles and detergent micelles (6, 7, 8). Chemically cross-linked αS in neuroblastoma cell homogenates was also found in vesicle fractions by flotation centrifugation (9). Fractionated brain extracts, however, only revealed a weak association of αS with synaptic vesicles (10, 11). Using a combination of fluorescence recovery after photobleaching and immunostaining approaches, Fortin *et al.* (12) reported that αS exists in neurons in both cytosolic and membrane-associated forms. These studies are consistent with a model of αS as an aqueously soluble protein that can transiently interact with vesicular membranes in a context-dependent manner. The cellular equilibrium between soluble and membrane-associated αS is expected to be finely balanced and tightly regulated. Both familial PD αS missense mutations and engineered variants were shown to shift the balance, eventually leading to misfolding, insolubility, inclusion formation, and cell toxicity (13, 14, 15, 16).

Besides αS, the human synuclein protein family consists of β-synuclein (βS) and γ-synuclein (γS). No studies have directly associated βS or γS with a synucleinopathy (17, 18, 19). However, occasional reports have identified βS and/or γS in synucleinopathy lesions, albeit not classical Lewy bodies (20, 21, 22). γS, unlike αS and βS (23), is not prevalent in the central nervous system (24, 25). Compared with αS, βS and γS were found to be less membrane-associated in a study that associated membrane binding rather than aggregation propensity with synuclein toxicity (26). The exact cytosol:membrane distribution of the synucleins in human neurons, however, remains unsettled, both in absolute and in relative terms.

We have now developed a simple and reproducible method employing digitonin-based sequential extraction of first cytosolic and then membrane-associated proteins, which readily enables us to characterize the cytosol:membrane distribution of synuclein proteins under basal and experimental conditions. We reasoned that centrifugal force and detachment of neurons from culture dishes may lead to rupture of membrane and may eventually influence our assessment. Thus, we adapted an on-plate, centrifugation-free procedure. Using this method and employing glyceraldehyde 3-phosphate dehydrogenase (GAPDH; largely cytosolic) and calnexin (transmembrane) as controls, we show that endogenous αS and βS in primary cortical neurons indeed seem to exist in an equilibrium of soluble and membrane-associated forms. αS was found to be more strongly membrane-associated than βS, while both synucleins exhibit a pronounced temperature dependence of their membrane interactions: body temperature promotes cytosolic localization up to 50% (αS) or >60% (βS), lower temperatures are associated with predominant membrane association of the synuclein proteins. Human wild-type (wt) αS expressed in rat neurons behaved similarly to the endogenous protein while familial-PD (fPD)-linked αS A30P was found to be largely cytosolic, in line with previous studies that had used orthogonal methods (27).

## Results

### Digitonin-based, centrifugation-free sequential extraction to achieve minimally disruptive cytosol:membrane protein separation

αS has been demonstrated to exhibit several aspects of dynamic behavior in its natural cellular environment, as reviewed recently by Yeboah *et al.* (28). In the present work, we sought to study the extent of αS-membrane interaction in a minimally disruptive way. As a model system we chose primary cortical rat neurons because they i) are rich in αS, ii) are easy to culture, iii) are readily available in large amounts for extensive optimization experiments, and iv) possess mature synapses after ∼14 days of culture (29). To assess αS cytosol:membrane distribution *in situ*, *i.e.*, without lifting cells off the culture dishes, we turned to digitonin-based sequential extraction of cellular proteins. Previous work from the Edwards lab had taken advantage of the same principle: to study only membrane-associated αS by fluorescence microscopy, the authors removed cytosolic αS by permeabilizing αS-expressing HeLa cells with digitonin before fixation (11). It is widely accepted that digitonin at low concentrations selectively permeabilizes the plasma membrane, thereby releasing soluble, cytoplasmic proteins (30).

### Sequential extraction of control proteins

**Figure 1:**
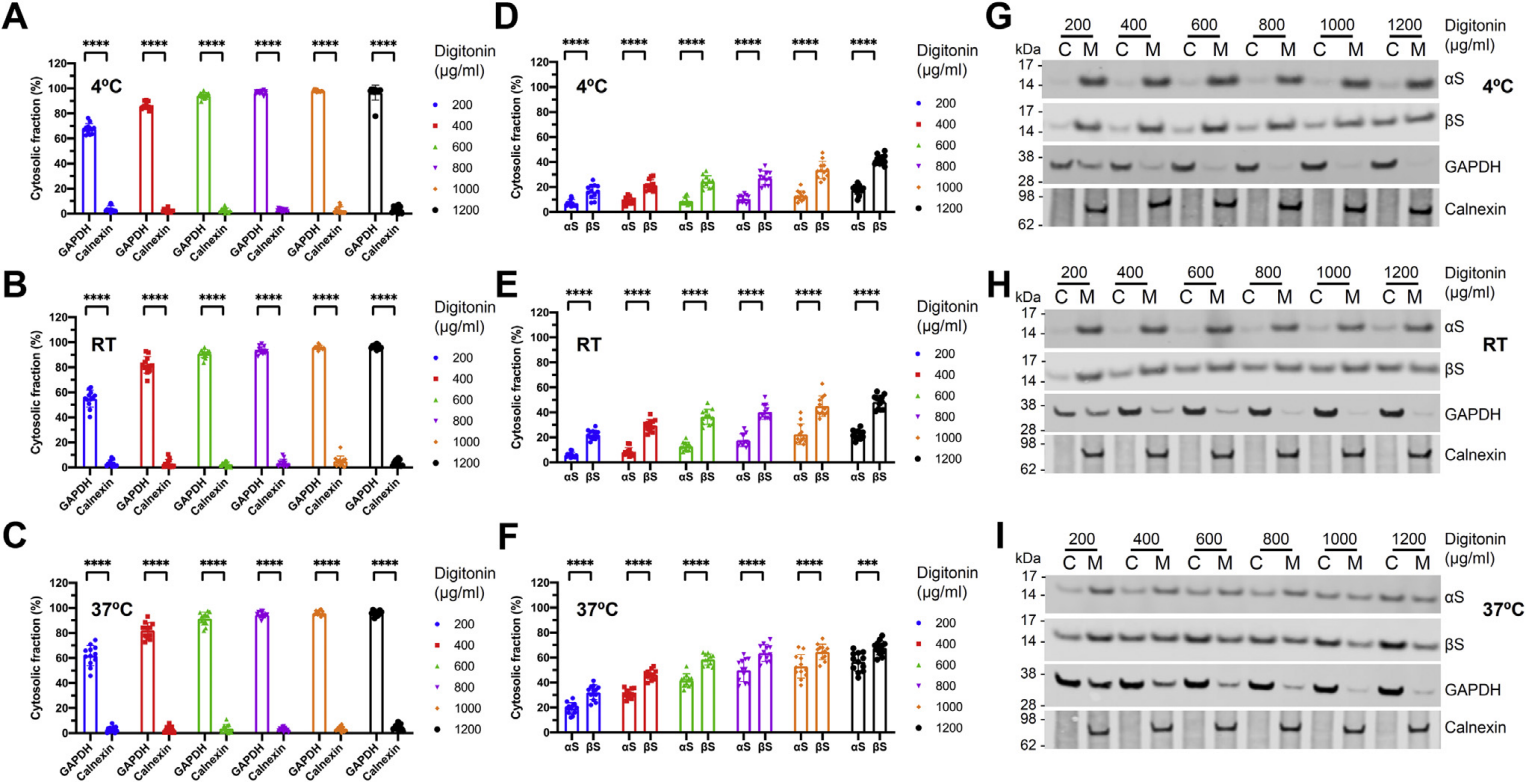
**Temperature-dependent digitonin-based sequential extraction of αS and βS.***A*–*C*, control proteins GAPDH (cytosol) and calnexin (membrane) were sequentially extracted, analyzed by western blotting, and cytosol:membrane ratios were calcuated. The temperatures during the extraction process were: *A*, 4 °C; *B*, RT; *C*, 37 °C. Digitonin concentrations of the first extraction step (cytosol) were as indicated in the legend; in the second extraction step (membrane) 0.5% Triton-X-100 was used. *D*–*F*, αS and βS were analyzed analogous to *A*–*C*. *G*–*I*, representative western blot images to the quantifications shown in *A*–*F* (C, cytosol and M, membrane). N = 3 independent experiments, performed on different days in n = 4 independent wells (total n = 12). ∗∗∗*p* < 0.001 and ∗∗∗∗*p* < 0.0001.

We decided to pursue a two-step biochemical sequential extraction strategy. In the first step, we extracted the soluble proteins (“cytosol”) by gently permeabilizing the plasma membrane, but not internal membranes such as endoplasmic reticulum or vesicle membranes. To achieve optimal stringency, we employed a wide range of digitonin concentrations (200–1200 μg/ml) and tested them under three different temperatures: 4 °C, room temperature (RT), and 37 °C (Fig. 1). The resulting cytosolic lysates were collected. In the second step, we incubated the cells in a buffer containing 0.5% of the detergent Triton-X-100, thereby largely extracting membrane proteins (“membrane”). The resulting membrane lysates were collected. Next, cytosolic and membrane lysates were analyzed by western blotting. To confirm the identity of the fractions, we employed the transmembrane control protein calnexin as well as GAPDH, which is widely used as a cytosolic marker (*e.g.*, Fig. 2*A* in ref. (31)). Under all conditions tested, calnexin was detected exclusively in the membrane fractions, as expected (Fig. 1, *A*–*C* and bottom panels in Fig. 1, *G*–*I*). In contrast, GAPDH was not solely detected in cytosolic fractions: at lower digitonin concentrations (200, 400, 600 μg/ml), relevant amounts were found in the membrane fraction (Fig. 1, *A*–*C*, and third panel from the top in Fig. 1, *G*–*I*). This either indicated incomplete extraction or could be explained by reports on GAPDH functions beyond its role as a cytosolic glycolytic enzyme (reviewed in ref. (32)). However, at 800 μg/ml and above, GAPDH was extracted to near completion, and the remaining contaminations in the membrane fraction appeared negligible for our purpose (it should be noted that in the first extraction step, cytosolic proteins will be highly enriched in the large extracellular volume, but a portion will also remain present in the small intracellular volume). Another glycolytic enzyme, phosphoglycerate kinase 1 behaved similarly to GAPDH (not shown).

### αS membrane association is temperature-dependent

Importantly, the calnexin and GAPDH extraction patterns did not obviously differ between different temperatures (Fig. 1, *A*–*C*). For αS, in contrast, we observed pronounced temperature-dependent effects: at 4 °C and RT, αS was largely detected in membrane fractions; even at high digitonin concentrations (800–1200 μg/ml), less than 20% of αS was cytosolic (Fig. 1, *D* and *E* and first panels from the top in Fig. 1, *G* and *H*). At 37 °C, however, a shift from membrane to cytosolic fractions was observed, and αS solubility increased to up to 50% (Fig. 1*F* and first panel from the top in Fig. 1*I*).

### βS membrane association is temperature-dependent as well and reduced relative to αS

We next compared the relative solubility of the two synuclein homologs that are prevalent in brain neurons, αS and βS (the third homolog, γS, exhibits a different tissue distribution). Unlike αS, βS has not been directly implicated in the pathogenesis of neurologic disorders. Similar to the extraction of αS, we found βS cytosol:membrane distribution to be temperature-dependent: at 4 °C, 20 to 40% of βS was extracted at low (200–600 μg/ml) and 50 to 60% at high (800–1200 μg/ml) digitonin concentrations (Fig. 1*D* and second panel from top in Fig. 1*G*). These numbers were only slightly higher at RT (Fig. 1, *E* and *H*). At 37 °C, however, we extracted 30 to 60% of βS at low (200–600 μg/ml) and 60 to 70% at high (800–1200 μg/ml) digitonin concentrations (Fig. 1, *F* and *I*). Thus, βS solubility was significantly higher than αS solubility under all conditions tested, as quantified in Figure 1, *D*–*F*.

### fPD-linked αS A30P is largely cytosolic

**Figure 2:**
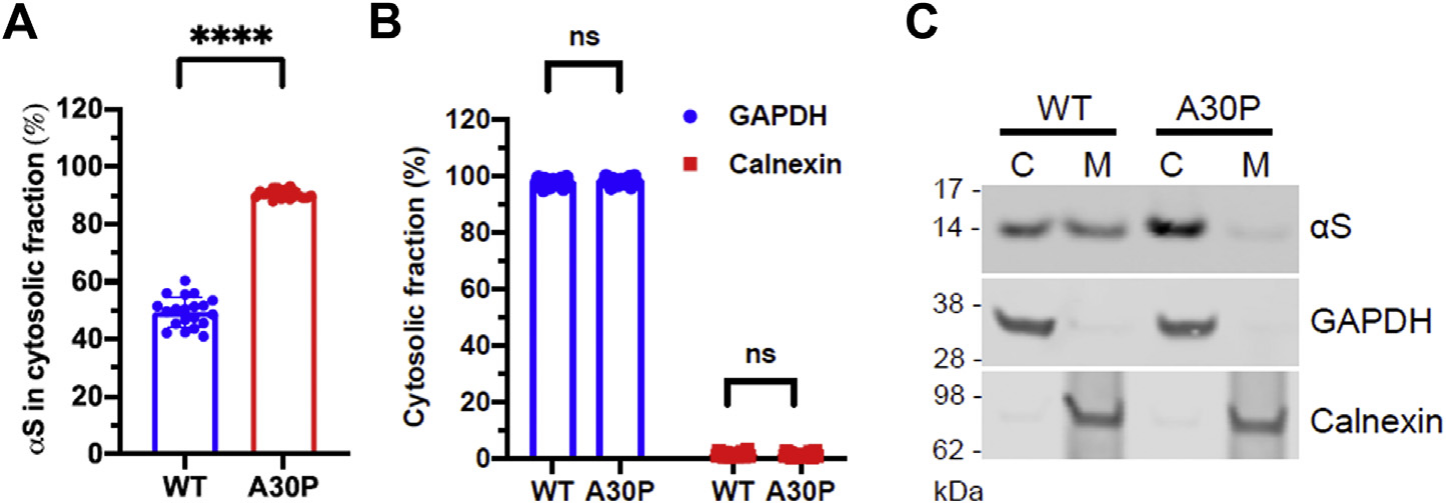
**The human αS A30P mutant is predominantly cytosolic.** Cytosolic and membrane fractions were sequentially extracted from cortical neurons expressing human wt or A30P αS at 37 °C (*A*). The solubility of control proteins GAPDH and calnexin is depicted in panel *B*. Representative western blot images to the quantification are shown in panel *C*. Digitonin concentration, 800 or 900 μg/ml. (C, cytosol and M, membrane). N = 3 independent experiments, performed on different days in n = 12 + 4 + 4 independent wells (total n = 20). ∗∗∗∗*p* < 0.0001. ns, not significant.

Employing what we considered the most relevant conditions (37 °C and 800–900 μg/ml digitonin, *i.e.*, the lowest digitonin concentrations that extracted GAPDH to near completion), we then tested the relative cytosol:membrane distribution of human αS wt and fPD-linked αS A30P. Transduced cultured rat cortical neurons were subjected to sequential extraction and immunoblotting using a human-specific antibody. Consistent with published reports that had used orthogonal methods (27), human αS A30P was observed to be largely cytosolic (cytosolic distribution: 90.4% ± 1.5%), exhibiting a distribution pattern similar to GAPDH (Fig. 2, *A*–*C*). Human wt αS, in contrast, partitioned 49.4% ± 5.2 between cytosolic and membrane fractions (Fig. 2, *A* and *C*), similar to endogenous rat αS (Fig. 1).

## Discussion

### Stringent sequential extraction of synuclein proteins in cultured neurons

Here, we systematically developed a stringent *in situ* sequential extraction method to study the cytosol:membrane distribution of synuclein proteins in primary rat cortical neurons. We employed a two-step approach: in a first step, we selectively permeabilized the plasma membrane using the mild detergent digitonin at various concentrations (200–1200 μg/ml) to extract cytosolic proteins. In a second step, we solubilized the more strongly membrane-associated proteins using the detergent Triton-X-100 at 0.5%. Cytosol (GAPDH) and membrane control proteins (calnexin) confirmed the validity of our approach. The results that we obtained for αS and βS are consistent with the notion that those proteins transiently/imperfectly interact with (vesicular) membranes (33).

### A surprising temperature dependence

When establishing our method, we found that αS and βS cytosol:membrane distribution strongly depends on temperature. At 4 °C and even at RT, αS, but also the homolog βS, was largely membrane-associated. Only at body temperature (37 °C) 50% or more of the synuclein proteins were recovered in the cytosolic fraction that was characterized by the presence of the soluble enzyme GAPDH and absence of the transmembrane protein calnexin. This observation once again highlighted the dynamic nature of cellular synuclein proteostasis: a temperature difference of only ∼12 °C (RT *versus* 37 °C) appears to have major consequences on cellular αS localization (and, related to that, folding). The implications on the design of biochemical and cell-biological studies of synuclein proteins are obvious: synuclein homeostasis is best studied at 37 °C while lower temperatures should be avoided, even for short periods of time. This means that standard protocols may have to be modified in the case of synuclein. For example, it is common to perform cell lysis on ice, which may lead to artifacts when αS is studied.

### Absolute and relative cytosol:membrane distribution of αS and βS in neurons

Our data do not permit a definite statement on the exact cytosol:membrane distribution of αS and βS in cultured neurons. There has been some debate about the lifetime of the membrane-bound αS state, and the reported numbers range from millisecond for purified recombinant αS in reductionist systems (34) to seconds or even minutes in cultured cells or animals (35, 36, 37). All these values, however, are below the duration of our first extraction step that recovers cytosol-enriched proteins within the timeframe of 15 min**.** αS partitioning is not expected to be “static” during the extraction, and the fact that the plasma membrane becomes “leaky” and additional, extracellular volume becomes available for soluble proteins may increase the observed fraction in the soluble phase (see “Nernst’s distribution law”). Consequently, our approach is more likely to detect false-positive cytosolic αS than false-positive membrane-associated αS. Out of the conditions that we have tested, it appears plausible to identify 37 °C and 800 μg/ml digitonin (the lowest digitonin concentration that extracted GAPDH to near completion) as most relevant and most likely to be close to physiology. Under these conditions, cytosolic distribution was 49.8% ± 9.0% for αS and 63.6% ± 6.6% for βS. These values are largely in agreement with the literature on synuclein as a transiently membrane-associated protein, as reviewed by Yeboah *et al.* (28). The relative distribution of αS and βS, *i.e.*, increased solubility of βS relative to αS, was a highly consistent finding throughout all conditions tested and, thus, is very likely to reflect the underlying biology. Along those lines, we characterized human αS A30P in Figure 2 as an abundantly cytosolic protein (cytosolic distribution: 90.4% ± 1.5%), while human αS wt behaved like a protein that populates both cytosolic and membrane-associated states to a largely similar extent (cytosolic distribution: 49.4% ± 5.2), similar to the endogenous rat protein (Fig. 1).

### Pathological implications of αS and βS solubility

The enhanced membrane association of αS relative to βS may explain certain aspects of αS pathogenicity. Indeed, Volles *et al.* (38) have reported, based on studying purified αS and αS-expressing yeast, that αS toxicity is directly linked to its membrane affinity, instead of its aggregation propensity. In the same study, the authors demonstrated that βS is more soluble and less toxic than αS (38). Our work, performed in cultured rodent neurons *in situ*, is consistent with this notion, supporting hypotheses of increased αS membrane association being a driver of its relative toxicity (38, 39). Increased membrane interaction of the PD-linked αS relative to the non-disease-relevant βS adds validity to therapeutic approaches aimed at reducing αS membrane interaction, such as SCD inhibition (40, 41, 42). It should not be ignored, however, that not all of the PD-linked αS missense mutations increase membrane interaction; at least A30P has been reported to reduce it (27), and our own data confirm this notion (Fig. 2). A simple model in which αS membrane interaction is either entirely “good” or entirely “bad” would not be consistent with the literature that has highlighted both excess membrane interaction (15, 39) and excess solubility (43) as potential starting points of αS-related pathology. Our new, stringent approach to biochemically testing synuclein membrane interactions in cultured neurons with minimal perturbation of the system promises to shed light on the role of transient membrane binding on the pathobiology of αS point mutations as well as the effects of genetic and environmental factors on αS homeostasis. Membrane interactions of αS are expected to be upstream of its proteinaceous aggregation. Thus, our work has important implications for assessing the ability of novel therapeutic strategies to maintain or reestablish normal αS cytosol:membrane interactions as a key aspect of αS proteostasis.

## Experimental procedures

### Plasmids

αS-wild-type or A30P lentiviral plasmids were generated as follows: first, the EF1α promoter sequence in pLVX-EF1α-IRES-mCherry (from TaKaRa) was replaced by human synapsin promoter, and then mCherry coding sequence was removed. The resultant parental plasmid (pLVX-SPΔ) was used to clone synthetic cDNA sequence coding for human αS wild-type or A30P variant into SpeI/NotI restriction sites. The expression of transgene is driven by human synapsin promoter.

### Lentivirus production

293-T cells were transfected with αS wt or A30P plasmids along with pMD2.G and psPAX2 (packaging plasmids: Addgene #12259 and #12260, respectively). Culture supernatant containing viral particles was further purified/concentrated by ultracentrifugation.

### Antibodies

As primary antibodies we used mAb Syn1 to detect rat endogenous αS (Becton-Dickinson), mAb MJFR1 to detect only human αS (Abcam), mAb EP1537Y to βS (Abcam), mAb 6C5 to GAPDH (Santa Cruz), and pAb C4731 to Calnexin (Sigma). Secondary antibodies were anti-rabbit Fluorescent LiCor IRDye 800CW and anti-mouse Fluorescent LiCor IRDye 700DX.

### Primary neuron cultures

Primary neurons were cultured from E18 Sprague-Dawley rats (Charles River, Wilmington, MA). Rats were euthanized with CO_2_ followed by bilateral thoracotomy. Embryonic cortices were isolated and dissociated with trypsin/EDTA and trituration. 250,000 cells were plated on poly-D-lysine coated 24-well plates and cultured in neurobasal medium supplemented with B-27, 2 mM GlutaMAX. Half of the medium was replaced every 4 days. For lentiviral transduction, DIV9 neurons were transduced with αS wt or A30P virus at MOI 5. Institutional animal work protocols were followed.

### Sequential protein extraction

DIV18–DIV21 neurons were used throughout. The on-plate sequential extraction of cytosolic and membrane-bound proteins was carried out as follows in 24-well plates: 1) neurons were rinsed once with HBSS. 2) In total, 125 μl of buffer “cytosol” (10 mM PIPES pH 7.4, 100 mM NaCl, 300 mM sucrose, 5 mM MgCl_2_, 5 mM EGTA) supplemented with the respective concentration of digitonin (D141, Sigma) and protease inhibitors was added to the wells. Note: 50 mg/ml digitonin stock was prepared freshly. 3) The plates were incubated at the respective temperature undisturbed for 15 min. The resultant cytosolic protein fraction was carefully collected into a 1.5 ml tube. 4) Subsequently, 125 μl buffer “membrane” (10 mM PIPES pH 7.4, 100 mM NaCl, 300 mM sucrose, 5 mM MgCl_2_, 5 mM EGTA, 0.5% Triton-X-100 and protease inhibitors) was added to the wells and incubated for 15 min at the respective temperature. 5) The resultant membrane fraction was carefully collected into a 1.5 ml tube.

### Gel electrophoresis and immunoblotting

Samples were prepared for electrophoresis by dilution with the respective lysis buffer, addition of 4X NuPAGE LDS sample buffer supplemented with 1.25% β-mercaptoethanol, and boiling for 5 min. Samples were electrophoresed on NuPAGE 4 to 12% Bis-Tris gels with NuPAGE MES-SDS running buffer and SeeBlue Plus2 molecular weight marker (all by Invitrogen) at 140 V and transferred in the iBlot 2 system (Invitrogen) to nitrocellulose membranes (iBlot 2 NC regular stacks; IB23001). Membranes were fixed for 10 min in 0.4% paraformaldehyde (in PBS). Nitrocellulose membranes were blocked in blocking buffer (5% milk in TBST) for 1 h and incubated in primary antibody in blocking buffer overnight at 4 °C. Membranes were washed 5 × 5 min in TBST. Secondary antibodies were prepared in the blocking buffer and incubated for 1 h at RT. Membranes were washed 5 × 5 min in TBST and scanned (Odyssey CLx, Li-Cor).

### Statistical analyses

We performed paired *t*-test or two-way ANOVA and Sidak’s multiple comparisons test using GraphPad Prism Version 8 following the program’s guidelines. Normal distribution and equal variance were observed for all values. Graphs include ± SD. Criteria for significance were: ∗∗∗*p* < 0.001 and ∗∗∗∗*p* < 0.0001. Sufficient experiments and replicates were analyzed to achieve statistical significance, and these judgments were based on earlier, similar work.

## Data availability

All data will be made available upon request. Please contact N.R. (nramalingam@bwh.harvard.edu) or U.D. (udettmer@bwh.harvard.edu).

## Conflict of interest

The authors declare that they have no conflicts of interest with the contents of this article.
